# Guiqi Baizhu Decoction Alleviates Radiation Inflammation in Rats by Modulating the Composition of the Gut Microbiota

**DOI:** 10.1155/2020/9017854

**Published:** 2020-10-19

**Authors:** Li-Ying Zhang, Ting Zhou, Yi-Ming Zhang, Xiao-Min Xu, Yang-Yang Li, Kong-Xi Wei, Jin-Peng He, Nan Ding, Heng Zhou, Yong-Qi Liu

**Affiliations:** ^1^Provincial-Level Key Laboratory for Molecular Medicine of Major Diseases and the Prevention and Treatment with Traditional Chinese Medicine Research in Gansu Colleges and Universities, Gansu University of Chinese Medicine, Lanzhou 730000, China; ^2^Key Laboratory of Space Radiobiology of Gansu Province & Key Laboratory of Heavy Ion Radiation Biology and Medicine of Chinese Academy of Sciences, Institute of Modern Physics, Chinese Academy of Sciences, Lanzhou 730000, China; ^3^Key Laboratory of Dunhuang Medicine and Transformation at Provincial and Ministerial Level, Gansu University of Chinese Medicine, Lanzhou 730000, China

## Abstract

The gut microbiota is important in metabolism and immune modulation, and compositional disruption of the gut microbiota population is closely associated with inflammation caused by ionizing radiation (IR). Guiqi Baizhu decoction (GQBZD) is a medicinal compound used in traditional Chinese medicine with anti-inflammatory and antioxidation effects, especially in the process of radiotherapy. However, the effect of GQBZD on reducing the damage to the normal immune system in radiotherapy remains unclear. Here, we show that GQBZD reduces body weights, water intake, food intake, diarrhea level and quality of life score, and inflammation and enhances immunity function in rats treated with X-ray radiation. Meanwhile, our data indicate that GQBZD not only reverses IR-induced gut dysbiosis as indicated change of *α*-diversity and *β*-diversity of microbiota, the composition of *Desulfovibrio*, *Bacteroides*, and *Parabacteroides*, except for *Roseburia* and *Lachnoclostridium*, but also maintains intestinal barrier integrity and promoting the formation of short-chain fatty acids (SCFAs). GQBZD can also reduce the level of phosphorylation P65 (p-P65). Our results demonstrate that GQBZD can significantly alleviate the inflammatory responses and improve the immune damage against IR, and may be used as prebiotic agents to prevent gut dysbiosis and radiation-related metabolic disorders in radiotherapy.

## 1. Introduction

Radiotherapy plays an important role in the treatment of both malignant and benign cancers for years [1]. However, its toxic effect to the surrounding normal healthy tissue remains a major drawback. Evidence has shown that the widespread use of ionizing radiation (IR) can cause high incidence of radiation-induced side effects in up to 75% during the process of radiotherapy [2]. The most common IR exposure damage effects are bone marrow inhibition and gastrointestinal toxicity [3, 4]. Mechanisms of host damage caused by IR have been studied, and particularly, gut microbiota's responsiveness is a key part of IR [5]. It has been demonstrated that the gut microbiota modulates radiation therapy and chemotherapy efficacy through “TIMER” mechanisms, which indicates translocation, immunomodulation, metabolism, enzymatic degradation, and reduced diversity, but mechanism details of this response and the prevention are limited.

The human gut microbiota consists of trillions of microbial cells and thousands of species of bacteria. Various internal and external environmental factors of the human body, including radiation, affect the balance of gut microbiota [6]. David et al. found that ^16^O radiation induces multimodal responses in the mouse gut microbiome [7]. The microbiota diversity and composition were changed at the functional level, while the phosphatidylinositol signaling could be linked to dose-dependent changes in the abundance of specific taxa. Zhang and Steen also shows that gut microbiome is very sensitive to IR and can be used as radiation biomarkers [8]. The gut microbiota has increasingly been recognized as a key contributor to human health, and an imbalance in the colonic microbiota might underlie many human diseases, such as diarrhea, immune hypofunction, and inflammatory reaction of intestine. Therefore, it is necessary to keep intestinal flora balance.

Most of the traditional Chinese medicine (TCM) produces effect by oral administration, which has good therapeutic effect against the illness. However, it is difficult to explain the detailed mechanism on TCM. Evidences have shown that TCM can alleviate diseases by regulating gut microbiota. Chang et al. indicate that *G. lucidum* and its high molecular weight polysaccharides can prevent gut dysbiosis and obesity-related metabolic disorders in obese individuals [9]. Alrafas et al. demonstrate that resveratrol modulates the gut microbiota to prevent murine colitis development through induction of Tregs and suppression of Th17 cells [10]. The traditional Chinese herbal formula, Gegen Qinlian decoction (GQD), can alleviate type 2 diabetes and enrich the amounts of beneficial bacteria, such as *Faecalibacterium* spp. [11]. Chaihu-Shu-Gan-San (CSGS) formulas may be used as prebiotics to modulate gut microbiota structure and take the protective effects on the host metabolic phenotype of the gut microbiota dysbiosis rats [12]. Huangqin decoction (HQD) could ameliorate dextran sulphate sodium (DSS)-induced colitis through alteration of the gut microbiota [13].

Guiqi Baizhu decoction (GQBZD), an herbal medicine containing seven active compounds, consists of *Astragalus membranaceus*, *Rhizoma Atractylodis Macrocephalae*, *Angelica*, *Paeonia lactiflora*, *dried tangerine peel*, *Rhubarb*, and *Licorice*, has been widely used in China as traditional treatment for many diseases. This herb possesses a number of beneficial therapeutic properties such as cytoprotective, antioxidative, anti-inflammatory, and antitumor activities. Clinically, GQBZD was used to reduce proinflammatory factor expression and the release of serum tumor markers and improve the life quality of gastric cancer patients [14]. Wang et al. proved that GQBZD has an attenuated effect on cisplatin-induced kidney injury, and its mechanism may be related to improve blood activity and reduce apoptosis of kidney tissue cells [15]. However, it is still unknown whether GQBZF can prevent radiation and what is its detail mechanism. In this study, using the high‐throughput 16S rRNA gene sequencing, we identified specific fecal microbial signatures in rats with radiation and sought to elucidate potential biomarkers or mechanistic principle show that the gut microbiota dysbiosis may impact the pathogenesis of radiation, and to investigate the protective effect of GQBZD on radiation-induced enteritis and its relationship with gut microbiota. The results maybe provide useful information about the therapeutic value of microecological preparation for radiation.

## 2. Materials and Methods

### 2.1. Animal

SD rats (6 weeks of age to 8 weeks of age, thirty males and thirty females) were purchased from Lanzhou Veterinary Research Institute, Chinese Academy of Agricultural Sciences. The animal certificate number is 62000600000293. All animal experiments reported in this study were approved by the Ethics Committee of Gansu University of Chinese Medicine. All animals housed in a controlled room (temperature, 25 ± 2°C; relative humidity, 45–60%; lighting cycle, 12 h/d; 06:30–18:30 for light) and had free access to food and drinking water during the experimentation.

### 2.2. Radiation

X-ray radiation was generated by X-RAD-225 X-ray source (Precision, North Branford, Connecticut, USA). The dose rate was about 200 cGy/min (225.0 kV, 13.3 mA). The total absorbed dose was 6 Gy. All irradiations were performed at room temperature.

### 2.3. Preparation and High-Performance Liquid Chromatography (HPLC)-Based Analysis of FL on the GQBZD

The TCM formula in our study was Chinese medicine decoction-free granules, named GQBZD, composed by seven herbs, namely: Huangqi (*Astragalus membranaceus*), Baizhu (*Rhizoma Atractylodis Macrocephalae*), Danggui (*Angelica*), Baishao (*Paeonia lactiflora*), Chenpi (*dried tangerine peel*), and Dahuang (*Rhubarb*), Gancao (*Licorice*), and the dosage, respectively, are 4.0 g, 3.3 g, 3.3 g, 10.0 g, 6.0 g, 8.6 g, and 6.0 g each pair of medicine. All components were prepared by simplification-free intelligent particle machine (Yifang pharmaceutical limited liability company, Guangdong, China) and purchased from the Affiliated Hospital of Gansu University of Chinese Medicine (prescription number: 2019194794).

Five samples were randomly selected, and high-performance liquid chromatography (HPLC) was used to detect the level of major components in the abovementioned GQBZD per dose, named *Astragaloside IV*, *Paeoniflorin*, and *Hesperidin*. HPLC analyses were performed on a Shimadzu liquid chromatography system (Shimadzu LC-2030, Kyoto, Japan) equipped with an evaporative light detector (Shimadzu 228-4511538, Kyoto, Japan). The chromatographic separation of the analytes was performed on a SinoChrom ODS-BP C18 analytical column (LC Column, 250 mm × 4.6 mm, 5 *μ*m). The sample injection volume was 10 *μ*L. An evaporative light-scattering detector was used to detect *Astragaloside IV*, and an ultraviolet detector was used to detect *Paeoniflorin* and *Hesperidin*. The wavelength of the *Astragaloside IV*, *Paeoniflorin*, and *Hesperidin* detectors was 270 nm, 230 nm, and 283 nm separately. The standard of *Astragaloside IV* (110781–201616, Beijing, China), *Paeoniflorin* (110736–201842, Beijing, China), and *Hesperidin* (110721–201115, Beijing, China) was purchased from the National Institutes for Food and Drug Control. The ratio of standard of *Astragaloside IV*, *Paeoniflorin*, *Hesperidin*, and methanol was 0.5 mg : 1 mL, 40 *μ*g : 1 mL, and 30 *μ*g : 1 mL.

### 2.4. Groups

Sixty SD rats were randomly assigned into Ctrl, Drug, IR, LD, MD, and HD groups (*n* = 10 each group). Rats in the Ctrl group were lavaged daily with saline. Rats in the IR group were treated with 6 Gy of X-ray irradiation and lavaged daily with saline. Rats in the LD group were treated with 6 Gy of X-ray irradiation and lavaged daily with low dose of GQBZD (4.1 g/kg). Rats in the MD group were treated with 6 Gy of X-ray irradiation and lavaged daily with middle dose of GQBZD (8.2 g/kg). Rats in the HD group were treated with 6 Gy of X-ray irradiation and lavaged daily with high dose of GQBZD (16.4 g/kg). Rats in the Drug group were treated similarly to the MD group (8.2 g/kg) but without irradiation. Rats were lavaged daily with saline and GQBZD at corresponding doses, respectively, for seven consecutive days.

### 2.5. Hematoxylin and Eosin (HE) Staining

Colon tissues were fixed with 4% paraformaldehyde at room temperature for 24 hours. The samples were embedded in paraffin, dehydrated, then sectioned at a 5 *μ*m thickness. The sections were stained with hematoxylin and 0.5% eosin for 5 and 3 minutes, respectively. Tissue sections were observed under a light microscope. Biological verifications were performed six times in each group.

### 2.6. ELISA Test

The expression of cytokines and inflammatory-associated proteins including IL-4, IL-6, IL-10, TNF-*α*, sIgA, PKC, HSP, ALP, and DAO in each group at different time points was detected by the ELISA test. The specific operation method was carried out following the manufacturer's instructions. Biological verifications was performed six times in each group.

### 2.7. Microbiota Profiling

Before the mice were sacrificed, the mice were fixed and their tails were lifted. We gently pressed the mouse's lower abdomen with our fingers and collected fresh feces in several sterile EP tubes with corresponding numbers. The EP tubes were stored at −80°C and transported with dry ice.

Total genome DNA from fecal samples was extracted for amplification using specific primer with the barcode (16S V3 + V4). Paired end sequencing was performed on the Illumina MiSeq Platform, and a phylogenic tree and OTU table were obtained from the Mothur Bayesian classifier. Sequencing libraries were generated and analyzed according to Zhang et al.'s previous study [16]. Principal coordinate analysis (PCoA) was performed to get principal coordinates and visualize from complex, multidimensional data. Observed-species, Shannon, Simpson, Chao1, and ACE are used to evaluate the complexity of species diversity [17]. OTUs were further used for genome prediction of microbial communities by PICRUST [18]. Biological verifications were performed six times in each group (three females and three males in each group).

### 2.8. Western Blot

Total protein colon samples were extracted using RIPA buffer (P0013C, Beyotime, Shanghai, China), and 15 *μ*g proteins were separated by 10% SDS-PAGE electrophoresis and transferred to a methanol-activated PVDF membrane (GVPPEAC12, Millipore, Bellerica, USA). The membrane was blocked for 1 h in PBST containing 5% milk and subsequently probed with Occludin antibody (GTX114949, GeneTex, Texas, USA), ZO-1 antibody (Ab96587, Cambridge, UK), P65 antibody (YT3108, ImmunoWay, Texas, USA), phosphorylated P65 antibody (B4568, ImmunoWay, Texas, USA), and GAPDH antibody (ab9485, Abcam, Cambridge, UK), respectively, for 2 h. After 1 h incubation with goat-anti-rabbit HRP-conjugated secondary antibody (ab97051, Abcam, Cambridge, UK), the protein bands were detected with luminal reagent (Millipore, Pittsburgh, USA) and their relative intensities were quantified using Image J. Biological verifications were performed three times in each group.

### 2.9. Statistical Analysis

All statistical analyses were performed by using the one-way analysis of variance (ANOVA) to test homogeneity of variances via Levene's test and followed with Student's *t*-test (SPSS 21.0 software). Data are expressed as the mean ± SEM and denoted as follows: ns *P* > 0.05; ^*∗*^*P* < 0.05; ^*∗∗*^*P* < 0.01; ^*∗∗∗*^*P* < 0.001.

## 3. Results

### 3.1. The Contents of Main Components in GQBZD

**Figure 1:**
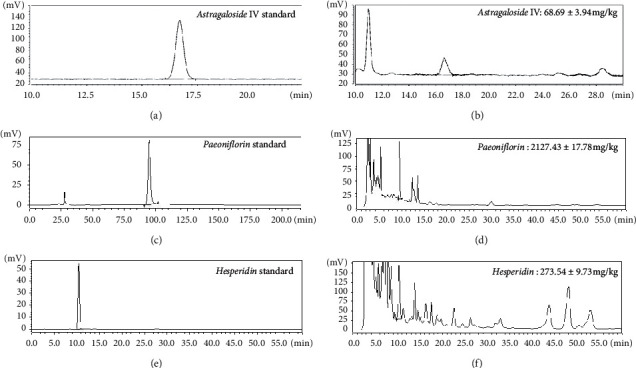
The contents of main components in GQBZD. The contents of main components in GQBZD were detected by high-performance liquid chromatography (HPLC). The results show the standard of *Astragaloside IV* (a), the sample of *Astragaloside IV* (b), the standard of *Paeoniflorin* (c), the sample of *Paeoniflorin* (d), the standard of *Hesperidin* (e), and the sample of *Hesperidin* (f) in GQBZD per dose.

The purposes of detecting the contents of the main components in GQBZD was to ensure the uniformity and standardization of the drugs. As shown in Figure 1, HPLC analyses revealed that the content of *Astragaloside IV* was 68.69 ± 3.94 mg/kg, the content of *Paeoniflorin* was 2127.43 ± 17.78 mg/kg, and the content of *Hesperidin* was 273.54 ± 9.73 mg/kg.

### 3.2. GQBZD Enhances the Life Quality and Immune Function of the Rats Exposed to IR

**Figure 2:**
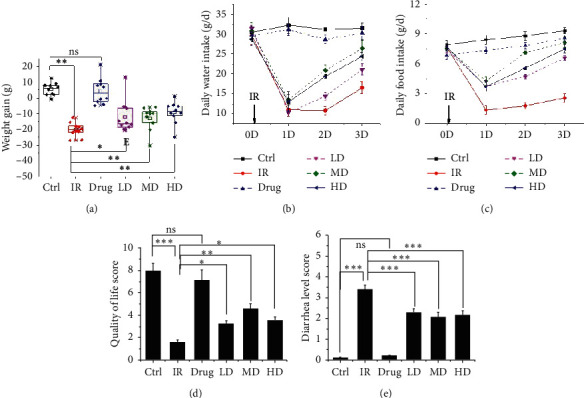
Effect of GQBZD on the state of affairs to rats in X-ray radiation. The results depict body weight (a), water intake (b), food intake (c), quality of life score (d), and diarrhea level score (e). Values are presented as mean ± SEM. Differences were assessed by ANOVA for multiple comparisons and denoted as follows: ^*∗*^*P* < 0.05; ^*∗∗*^*P* < 0.01; ^*∗∗∗*^*P* < 0.001; ns *P* > 0.05.

**Figure 3:**
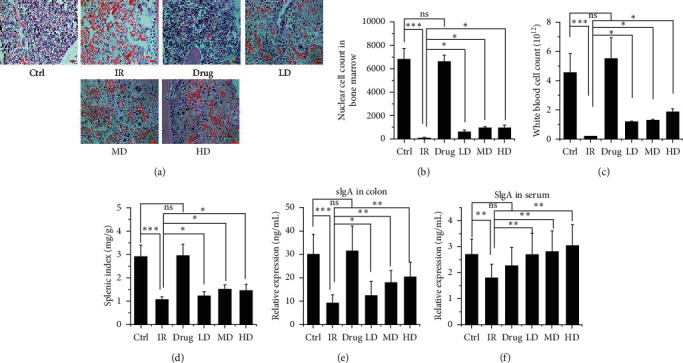
Effect of GQBZD on the immune function of X-ray radiation. Histopathological observations of the bone marrow stained with hematoxylin-eosin (a) were carried out under light microscopy with 40x magnification (scale bar 50 *μ*m). Nucleated cells in bone marrow were counted in microscope (b). White blood cell was counted (c), and splenic index was detected (d). Relative expression of sIgA in colon (e) and in serum (f) was assessed using the ELISA test. Values are presented as mean ± SEM. Differences were assessed by ANOVA for multiple comparisons and denoted as follows: ^*∗*^*P* < 0.05; ^*∗∗*^*P* < 0.01; ^*∗∗∗*^*P* < 0.001; ns *P* > 0.05.

To determine the effect of GQBZD on X-ray radiation rats, body weight, water intake, food intake, quality of life score, diarrhea level score, and the immune function were tested. As shown, X-ray radiation significantly decreased the body weight, water intake, food intake, quality of life score, and diarrhea level score (*P* < 0.05) (Figures 2(a)–2(e)) and X-ray radiation significantly decreased the nucleated cells in bone marrow, white blood cell in blood, splenic index, and the secretion of sIgA both in serum and in colon (*P* < 0.05) (Figures 3(a)–3(f)). GQBZD treatment tended to alleviate X-ray radiation-induced weight loss, water intake loss, and food intake loss (*P* < 0.05) (Figures 2(a)–2(c)), and GQBZD treatment alleviated the life quality and diarrhea level scores in X-ray radiation rats (Figures 2(d)–2(e)). In addition, GQBZD treatment alleviated X-ray radiation-induced immune function. The nucleated cells in bone marrow, white blood cell in blood, splenic index, and the secretion of sIgA in serum and in colon increased in the GQBZD group compared to the X-ray radiation group (*P* < 0.05) (Figures 3(a)–3(f)), whereas GQBZD enhances the life quality and immune function in rats treated by X-ray radiation.

### 3.3. GQBZD Alleviates the Inflammatory Response to X-Ray Radiation

**Figure 4:**
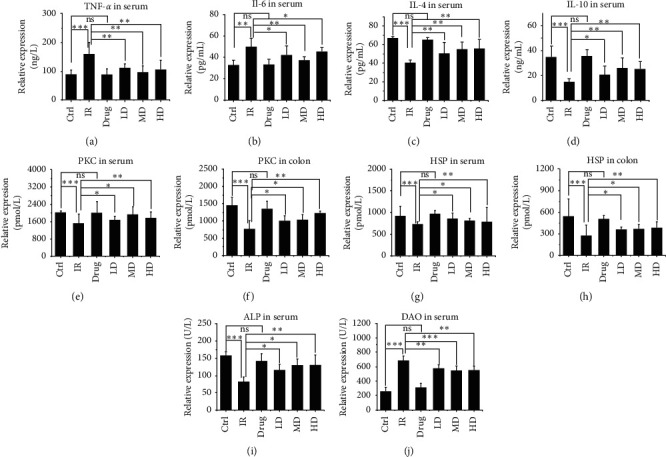
Effect of GQBZD on inflammatory cytokine and function protein expression in the serum and in colon in X-ray radiation. Relative expression of TNF-*α* (a), IL-6 (b), IL-4 (c), IL-10 (d), PKC in serum (e), PKC in colon (f), HSP in serum (g), HSP in colon (h), ALP in serum (i), and DAO in serum (j) was assessed using the ELISA test and in comparison with the X-ray radiation group. Values are presented as mean ± SEM. Differences were assessed by ANOVA for multiple comparisons and denoted as follows: ^*∗*^*P* < 0.05; ^*∗∗*^*P* < 0.01; ^*∗∗∗*^*P* < 0.001; ns *P* > 0.05.

To determine the effect of GQBZD on the immune system, the expression of inflammatory cytokines were detected. The results showed that X-ray radiation significantly decreased the secretion of IL-4, IL-10, HSP, PKC, and ALP and increased the expression of IL-6, TNF-*α*, and DAO (*P* < 0.05) (Figures 4(a)–4(j)), whereas GQBZD treatment changed the secretion of IL-4, IL-10, HSP, PKC, and ALP increased, and the expression of IL-6, TNF-*α*, and DAO decreased in GQBZD compared to the X-ray radiation group (*P* < 0.05), indicating that GQBZD treatment alleviates X-ray radiation-induced inflammatory response (Figures 4(a)–4(j)).

### 3.4. GQBZD Attenuates Radiation-Induced Colitis and Intestinal Damage

**Figure 5:**
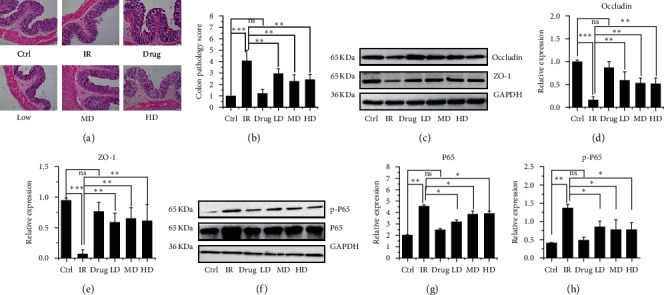
Effect of GQBZD on the inflammatory injury of intestinal in X-ray radiation rats. Histopathological observations of the colon stained with hematoxylin-eosin (a) were carried out under light microscopy with 40x magnification (scale bar 50 *μ*m). Colon pathology score was counted in microscope (b). Occludin ((c) and (d)), ZO-1 ((c) and (e)), P65 ((f) and (g)), and p-P65 ((f) and (h)) were tested by western blot and in comparison with the X-ray radiation group. Values are presented as mean ± SEM. Differences were assessed by ANOVA for multiple comparisons and denoted as follows: ^*∗*^*P* < 0.05; ^*∗∗*^*P* < 0.01; ^*∗∗∗*^*P* < 0.001; ns *P* > 0.05.

To determine the effect of GQBZD on the inflammatory injury of intestinal in X-ray radiation rats, HE stain of colon, protein Occludin, ZO-1, P65, and p-P65 were tested (Figures 5(a)–5(h)). The result showed that X-ray radiation significantly increased intestinal mucosal injury (Figures 5(a) and 5(b)) and the expression of P65 and p-P65 (Figures 5(f)–5(h)) in rats, while the expression of gap junction protein Occludin (Figures 5(c) and 5(d)) and ZO-1 (Figures 5(c) and 5(g)) downregulated (*P* < 0.001). GQBZD treatment tended to alleviate X-ray radiation-induced Occludin and ZO-1 loss and downregulated the expression of P65 and p-P65 (*P* > 0.05) (Figures 5(c)–5(e)).

### 3.5. GQBZD Reprograms the Gut Microbiota in Radiation Rats

**Figure 6:**
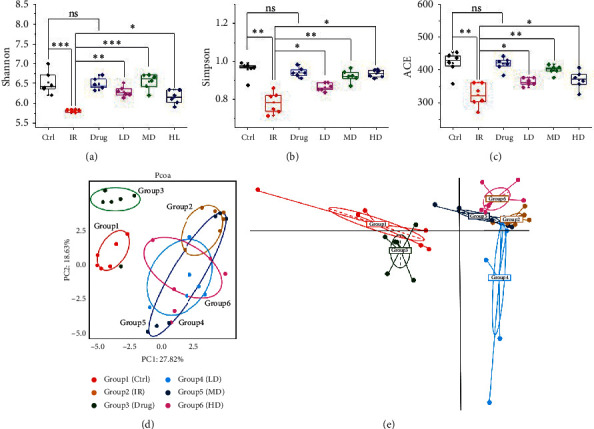
GQBZD improved gut microbiota diversity in X-ray radiation rats. (a) Shannon index in ɑ-diversity analysis. (b) Simpson index in ɑ-diversity analysis. (c) ACE index in ɑ-diversity analysis. (d) PCoA plot analysis from each sample. (e) PCoA space plot analysis from each sample. Values are presented as mean ± SEM, *n* = 6. Differences were assessed by ANOVA for multiple comparisons and denoted as follows: ^*∗*^*P* < 0.05; ^*∗∗*^*P* < 0.01; ^*∗∗∗*^*P* < 0.001; ns *P* > 0.05.

Gut microbiota is highly associated with inflammation; thus, we further determined fecal microbiota compositions by sequencing the bacterial 16S rRNA (*n* = 6). Shannon, Simpson, and ACE indexes were examined for the alpha-diversity of the microbiomes. Radiation rats exhibited a lower diversity of microbiota evidenced by the decreased Shannon, Simpson, and ACE indexes compared to controls (*P* < 0.05) (Figures 6(a)–6(c)). Interestingly, GQBZD treatment markedly increased Shannon and Simpson indexes (*P* < 0.01), suggesting an improvement in gut microbiota diversity in radiation rats. To evaluate overall differences in beta-diversity, we applied PCoA plot and PCA space diagram to weighted and unweighted UniFrac distance metric matrices generated for the sample set. Control and radiation rats presented a distinct clustering of microbial community structure, while the GQBZD group had a similar structure to that of the control rats (Figures 6(d)–6(e)).

**Figure 7:**
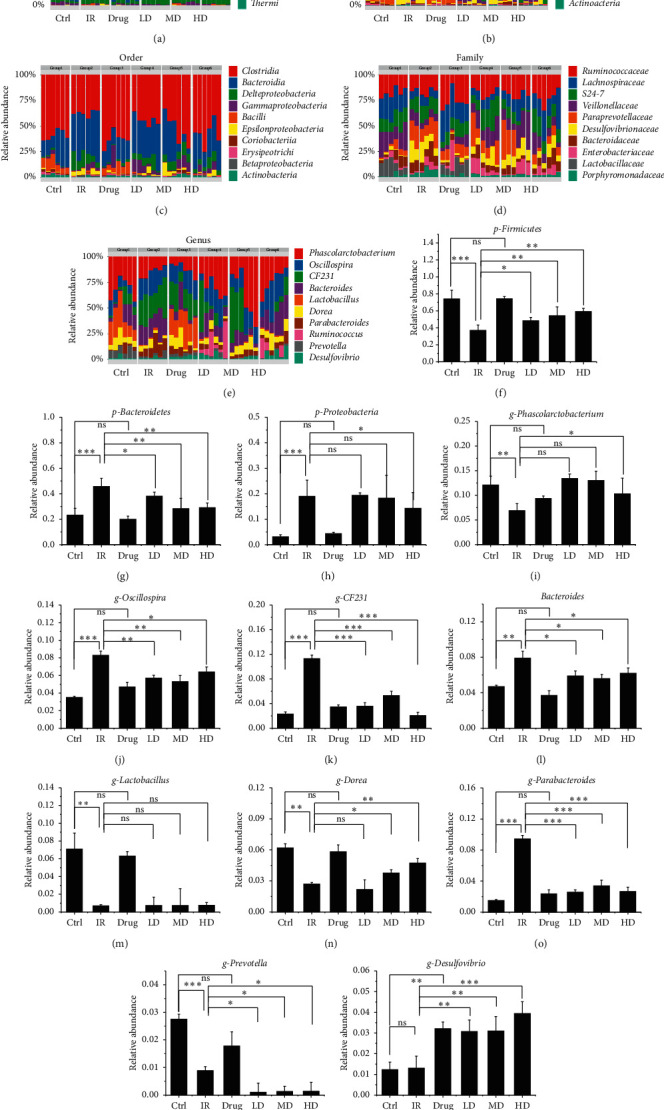
GQBZD improved gut microbiota in X-ray radiation rats. Microbiota compositions at the phylum level (a), at the class level (b), at the order level (c), at the family level (d), and at the genus level (e). The figures show bacteria with obvious changes at the genus level ((f)–(h)). The figures show bacteria with obvious changes at the genus level ((i)–(q)). Values are presented as mean ± SEM. Differences were assessed by ANOVA for multiple comparisons and denoted as follows: ^*∗*^*P* < 0.05; ^*∗∗*^*P* < 0.01; ^*∗∗∗*^*P* < 0.001; ns *P* > 0.05.

The overall microbial composition in each group differed at the phylum, class, order, family, and genus levels (Figures 7(a)–7(e)). The largest phylum represented in each dataset was *Firmicutes*, *Bacteroidetes*, and *Proteobacteria*. Radiation rats had a higher relative abundance of *Bacteroidetes* and *Proteobacteria*, but a lower relative abundance of *Firmicutes* and the ratio of *Firmicutes/Bacteroidetes* (*P* < 0.001), while GQBZD markedly reversed these alterations in *Firmicutes*, *Bacteroidetes*, and *Proteobacteria* and the ratio of *Firmicutes/Bacteroidetes* (Figures 7(f)–7(h)).

At the genus level, the top 10 genera represented in each dataset were *Phascolarctobacterium*, *Oscillospira*, *CF231*, *Bacteroides*, *Lactobacillus*, *Dorea*, *Parabacteroides*, *Ruminococcus*, *Prevotella*, and *Desulfovibrio*. Rats treated with IR had a higher relative abundance of *Oscillospira*, *CF231*, *Bacteroides*, and *Parabacteroides* (*P* < 0.05), and a lower relative abundance of *Phascolarctobacterium*, *Lactobacillus*, *Dorea*, and *Prevotella* (*P* < 0.05), but changed not in *Desulfovibrio* (*P* > 0.05). Fortunately, GQBZD could change most of the above indicators and it markedly reversed these alterations in *Phascolarctobacterium*, *Oscillospira, CF231*, *Bacteroides*, *Dorea*, *Parabacteroides*, and *Prevotella* (*P* < 0.05) but except in *Lactobacillus* (*P* > 0.05). An interesting thing was that *Desulfovibrio* responses to GQBZD but not IR, and it raised by the action of GQBZD drugs only (Figures 6(i)–6(q)).

### 3.6. GQBZD Adjusted Gut Microbiota Function and the Metabolites

The gut microbiota function and the metabolites were predicted by PICRUST analysis. Among them, radiation markedly reduced stress-tolerant and contains-mobile-elements, but enhanced potentially-pathogenic (*P* < 0.05) (Figures 8(a)–8(c)). GQBZD treatment reprogramed the stress-tolerant, contains-mobile-elements, and potentially-pathogenic (*P* < 0.05) (Figures 8(a)–8(c)). Metabolites of gut microbiome, such as acetic acid, propionic acid, and butyric acid, were detected by gas chromatography-mass spectrometry. Radiation markedly reduced acetic acid, propionic acid, and butyric acid, and GQBZD treatment reprogramed them (*P* < 0.05) (Figures 8(d)–78).

## 4. Discussion

Ionizing radiation (IR), a recognized proinflammatory agent, can cause oxidative stress, inflammation, p53 induction, DNA damage, mutagenesis, and oxidation of various molecules such as lipids and proteins [19–21]. IL-4, IL-6, IL-10, and TNF-*α* are representative inflammatory factors, PKC, HSP, ALP and DAO are products of stress reaction, and both of them are all sensitive to radiation and have response to radiation stress [21–23]. The current study demonstrated that IR (6 Gy) decreased the body weight gain, the number of nucleated cells in bone marrow, spleen index, and the screen of sIgA both in colon and serum, indicating immune function descent. Meanwhile, in this paper, X-ray radiation significantly decreased the anti-inflammatory factor secretion of IL-4 and IL-10, and increased the proinflammatory factor expression of IL-6, TNF-*α*, HSP, and PKC, whereas IR promoted the inflammation and alleviated immune function in rats. This is the same as Mukherjee et al.'s study [24]. GQBZD is a traditional Chinese Medicine. Studies from our laboratory and in clinic have shown its ability to reduce the symptoms associated with colitis in mice and crowd models, and GQBZD conferred meaningful prevention on inflammation in rats [14, 15]. It has been reported that gut microbiota plays a great role in the course of action of traditional Chinese medicine [25]. The anti-inflammatory, radio-resistance effects of TCM may be partly mediated by regulating the composition of gut microbes [26]. Many effective components of GQBZD have been proved to have antiradiation effect. Li et al. demonstrated that *Astragaloside IV* showed radio protective effects on the hematopoietic system in mice [27], and Liu et al. suggest that *Astragaloside IV* can antagonize radiation-induced brain cells senescence, and its mechanism may be related to p53-p21 and p16-RB signaling pathways of aging regulation [28]. Yue et al. found that *Paeoniflorin* enhances its sensitivity to radiotherapy by regulating MLH1 and MSH1 protein to enhance the DNA damage and repair ability of melanoma cells [29]. Santhanam et al. obtained that *Hesperidin* has the potential to be used as active ingredients in sunscreen and antiphoto aging formulations [30]. Hewage et al. found that *Hesperidin* shielded human keratinocytes from UVB radiation-induced damage and apoptosis via its antioxidant and UVB absorption properties [31]. In clinic, we also have observed that GQBZD is a safe and effective treatment for diarrhea of gastric cancer patients and that it can slightly alleviate diarrhea associated with irritable bowel syndrome in tumor. We, therefore, speculated that GQBZD's effects on the development of radiation might depend on gut microbiota.

Diarrhea is among the most common side effects of radiation therapy for malignant tumor [27, 32, 33]. There is evidence to suggest that changes in the gut microbial balance are involved in the onset of radiation-induced diarrhea: probiotics have been shown to prevent occurrence of radiation-induced diarrhea, and germ-free mice are more resistant to radiation enteritis [34]. In this study, we took advantage of the SD rat model of radiation that allowed localized injury to the rectum by abdominal irradiation which mimics human radiation proctitis. We discovered a significant *α*-diversity declining and *β*-diversity changing in colon contents collected from the radiation rats. Many research shows this is a common phenomenon in multiple animal models, such as in *Bactrocera dorsalis* [35], in the bank vole [36], in the Gottingen minipig and rhesus macaque models [37], and in mice and human [6, 7]. Besides, similar to the study of Casero et al. [7], a significant increase was revealed in the relative abundance of *Bacteroidetes* and *Proteobacteria* and decrease was revealed in the relative abundance of *Firmicutes* at phylum level in the IR rats, and GQBZD could reverse the changes. Dysbiosis of gut microbiota was typified by a bloom in the ratio of *Bacteroidetes* and *Firmicutes*. In this study, radiation rats had a higher relative abundance of *Bacteroidetes* and *Proteobacteria*, but a lower relative abundance of *Firmicutes* and the ratio of *Firmicutes/Bacteroidetes*, while GQBZD markedly reversed these alterations in *Firmicutes*, *Bacteroidetes*, *Proteobacteria* and the ratio of *Firmicutes/Bacteroidetes*. The largest genus represented in each dataset were *Desulfovibrio*, *Bacteroides*, *Parabacteroides*, *Lactobacillus*, *Roseburia*, and *Lachnoclostridium*. Radiation rats had a higher relative abundance of *Desulfovibrio*, *Bacteroides*, *Parabacteroides* but a lower relative abundance of *Lactobacillus*, *Roseburia*, *Lachnoclostridium* (*P* < 0.001), while GQBZD markedly reversed these alterations in *Desulfovibrio*, *Bacteroides*, *Parabacteroides*, and *Lactobacillus* (*P* < 0.01) but except in *Roseburia* and *Lachnoclostridium*.

Variation of the composition of intestinal flora is often accompanied with the changes of the function of intestinal flora and the difference to the metabolic production of intestinal flora. In this paper, the PICRUST analysis was used to predict the gut microbiota function and the metabolites. The results show that radiation markedly reduced the level of anaerobic, the function of stress-tolerant, and contains-mobile-elements decreased, but enhanced the level of aerobic and the possibility of potentially-pathogenic. GQBZD reprogramed the expression of them. The short-chain fatty acids (SCFAs), metabolites of gut microbial, have shown promise for treating inflammatory bowel disease [38], mediating intestinal barrier function [39] and increasing anti-inflammatory cytokine secretion [40]. In this paper, we take advantage of gas chromatography-mass spectrometry to test the expression of acetic acid, propionic acid, and butyric acid, important metabolites of the intestinal flora. The result demonstrated that radiation markedly reduced the level of acetic acid, propionic acid, and butyric acid, and GQBZD treatment reprogramed them. It has been reported that species of *Desulfovibrio* has the ability to reduce several toxic metals such as uranium, chromium, and iron [41], *Desulfovibrio* has the potential to generate energy (ATP) through electron transfer-coupled phosphorylation [42], and *Bacteroides* is the producer of butyric acid, induces IL-10 secreting B- and T-cells that prevent viral encephalitis [43]. It is an evidence that variation of the composition of the gut microbiota accompanied with the alteration of the function and the metabolic production of the gut microbiota, and GQBZD can partially relieve the changes of intestinal microecological components.

Intestinal homeostasis is maintained by a unique underlying inflammatory tone that is modulated by many pathways. Disorders of these pathways are associated with an increase in intestinal damage. Studies have shown that NF-*κ*B signaling in epithelium is an important homeostatic pathway regulating intestinal inflammatory response [44]. Intestinal epithelial specific conditions in knockout mice suggest that normalizing the NF-*κ*B pathway is essential for the survival and protection of colitis epithelial cells [45]. Interestingly, activation of the constitutive NF-*κ*B pathway may also lead to increased expression and injury of inflammatory mediators in intestinal epithelial cells [46]. In this work, we tested the level of P65 and the phosphorylation P65. The phosphorylation sites of P65 are located in the S276 regions of the N-terminal active region. When serine 276 is phosphorylated, P65 can be activated and the inflammation levels in the downstream increased in the IR group. GQBZD can downregulate the P65 phosphorylation. Our findings indicated that the potential applications of GQBZD in the treatment of radiation-induced systemic inflammation and metabolic syndrome might closely depend on its modulatory effect on gut microbiota. To confirm the results of GQBZD acting on gut microbiota, a more complete analysis of the microorganisms in the gut is suggested for further studies.

## 5. Conclusion

**Figure 9:**
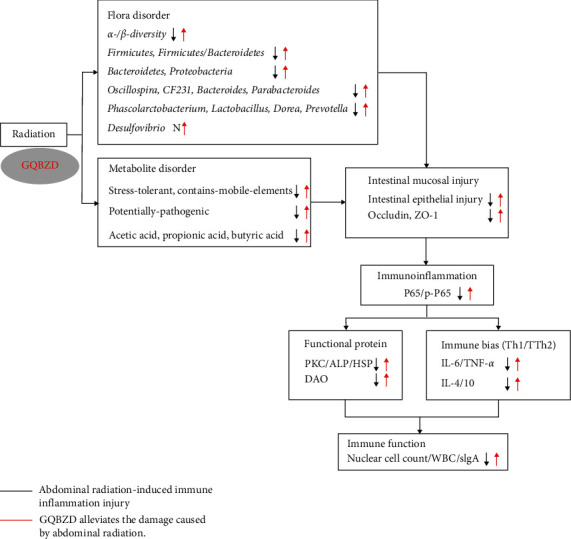
A proposed model of regulating intestinal microecology by GQBZD in rats exposed IR. Black arrows indicate that abdominal irradiation causes intestinal flora and their metabolite disorder, inflammatory damage, and immune function decline. Red arrows indicate that GQBZD alleviates the damage caused by abdominal radiation.

In this study, a proposed model of regulating intestinal microecology by GQBZD in rats was summarized (Figure 9). The NF-*κ*B signaling pathway is probably one of the mechanisms underlying the regulating intestinal microecology of GQBZD. In summary, rats treated with IR experienced change with the composition of the gut microbiota, and its metabolites and biological functions changed. This maybe leads to intestinal permeability increased and inflammatory level increased and immune decreased. Our results disclose that GQBZD alleviates radiation inflammation in rats by modulating the composition of the gut microbiota and suppresses P65 activation, as well as decreases the expression of the inflammatory factor. In this work, gut microbiota may be the target of GQBZD, but “who” GQBZ works for and “how” GQBZD works is an interesting topic worth further investigation.

## Figures and Tables

**Figure 8:**
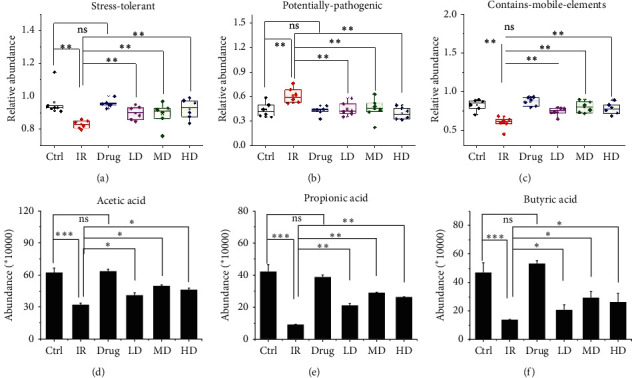
GQBZD adjusted gut microbiota function and metabolites. Stress-tolerant (a), potentially-pathogenic (b), and contains-mobile-elements (c) were predicted by BugBase. Acetic acid (d), propionic acid (e), and butyric acid (f) were detected by gas chromatography-mass spectrometry. Values are presented as mean ± SEM. Differences were assessed by ANOVA for multiple comparisons and denoted as follows: ^*∗*^*P* < 0.05; ^*∗∗*^*P* < 0.01; ^*∗∗∗*^*P* < 0.001; ns *P* > 0.05.

## Data Availability

Due to confidentiality agreements, supporting data can only be made available to bona fide researchers subject to a nondisclosure agreement. Details of the data and how to request access are available from data manager contact info at the Provincial-Level Key Laboratory for Molecular Medicine of Major Diseases and The Prevention and Treatment with Traditional Chinese Medicine Research in Gansu University of Chinese Medicine.
